# Phylogenetic Analysis of Dengue Virus in Bangkalan, Madura Island, East Java Province, Indonesia

**DOI:** 10.1155/2018/8127093

**Published:** 2018-02-15

**Authors:** Teguh Hari Sucipto, Tomohiro Kotaki, Kris Cahyo Mulyatno, Siti Churrotin, Amaliah Labiqah, Soegeng Soegijanto, Masanori Kameoka

**Affiliations:** ^1^Indonesia–Japan Collaborative Research Center for Emerging and Reemerging Infectious Diseases, Institute of Tropical Disease, Airlangga University, JI. Mulyorejo, Surabaya 60115, Indonesia; ^2^Center for Infectious Diseases, Kobe University Graduate School of Medicine, 7-5-1 Kusunoki-cho, Chuo-ku, Kobe, Hyogo 650-0017, Japan; ^3^Department of International Health, Kobe University Graduate School of Health Sciences, 7-10-2 Tomogaoka, Suma-ku, Kobe, Hyogo 654-0142, Japan

## Abstract

Dengue virus (DENV) infection is a major health issue in tropical and subtropical areas. Indonesia is one of the biggest dengue endemic countries in the world. In the present study, the phylogenetic analysis of DENV in Bangkalan, Madura Island, Indonesia, was performed in order to obtain a clearer understanding of its dynamics in this country. A total of 359 blood samples from dengue-suspected patients were collected between 2012 and 2014. Serotyping was conducted using a multiplex Reverse Transcriptase-Polymerase Chain Reaction and a phylogenetic analysis of E gene sequences was performed using the Bayesian Markov chain Monte Carlo (MCMC) method. 17 out of 359 blood samples (4.7%) were positive for the isolation of DENV. Serotyping and the phylogenetic analysis revealed the predominance of DENV-1 genotype I (9/17, 52.9%), followed by DENV-2 Cosmopolitan type* (7/17, 41.2%)* and DENV-3 genotype I* (1/17, 5.9%)*. DENV-4 was not isolated. The Madura Island isolates showed high nucleotide similarity to other Indonesian isolates, indicating frequent virus circulation in Indonesia. The results of the present study highlight the importance of continuous viral surveillance in dengue endemic areas in order to obtain a clearer understanding of the dynamics of DENV in Indonesia.

## 1. Introduction

Dengue is the most important mosquito-borne viral infectious disease in tropical and subtropical areas [1]. It is caused by an infection with any of the four distinct serotypes of dengue virus (DENV-1 to DENV-4). More than 390 million cases of dengue infection occur worldwide every year [2]. Dengue causes various clinical manifestations, ranging from dengue fever (DF) to more severe forms of the disease, such as dengue hemorrhagic fever (DHF) and dengue shock syndrome (DSS) [3].

Indonesia is one of the largest dengue endemic countries worldwide [4, 5]. Dengue occurred for the first time as an outbreak in Surabaya and Jakarta in 1968 [6, 7]. Dengue fever has spread to all regions of this country and the number of cases reported has been increasing. Dengue occurs in all 34 provinces in Indonesia annually and major outbreaks have been periodically reported [5]. The predominant viral type changes depending on the region and time. For example, in Surabaya, the second biggest city in Indonesia, the predominance of DENV-1 was reported from November 2008 until June 2013 [8], while that of DENV-2 was before November 2008 and after June 2013 until December 2015 [9]. The continuous monitoring of circulating serotypes is important because serotype or genotype shifts may be associated with increases in disease severity or dengue outbreaks [1].

Bangkalan is the biggest city in Madura Island, East Java province, Indonesia. It is located 37.8 km from Surabaya. The number of dengue cases in this city was previously reported to be higher than that in other cities in Madura Island [10]. The Ministry of Health reported an increase in the number of dengue cases: 333 cases occurred in 2009, 728 in 2010, 63 in 2011, 397 in 2012, 459 in 2013, and 277 in 2014. Many workers in Madura Island move to Jakarta or Surabaya for occupational purposes and may frequently return. Therefore, the high frequency of movement of residents in Bangkalan causes active viral movement. However, molecular information on dengue in Bangkalan has not yet been reported. Therefore, the phylogenetic analysis of DENV was performed in order to obtain a clearer understanding of its dynamics in Bangkalan.

## 2. Materials and Methods

### 2.1. Sample Preparation

A total of 359 blood samples were collected from patients diagnosed with dengue at the Syarifah Ambami Rato Ebu General Hospital, which is the largest hospital in Bangkalan city. Dengue was diagnosed by clinical manifestations, hematocrit, and platelet count. Viral antigen detection was not performed at the hospital. Signed informed consent by patients or their parents was obtained upon blood collection. This study was approved by the Ethics Committees of Airlangga University (Ethics Committee approval number: 24-934/UN3.14/PPd/2013) and the Kobe University Graduate School of Medicine (Ethics Committee approval number: 784).

### 2.2. Sample Collection and Virus Isolation

Blood samples were subjected to virus isolation as previously reported [8]. Briefly, serum specimens diluted in culture medium (1 : 10) were inoculated onto a Vero cell monolayer at 37°C under 5% CO_2_ for seven days. After three blind passages, cells were subjected to immunostaining with a* Flavivirus* group cross-reactive monoclonal antibody (D1–4G2; American Type Culture Collection, Manassas, VA) in order to examine the presence of viral antigens. Antigen-positive cells were subjected to RNA extraction using the QIAamp viral RNA Mini Kit (QIAGEN, Hilden, Germany). Viral antigen or gene detection in the sera was not performed prior to virus isolation, since the main purpose of the present study was to examine the phylogenetic relationship of Madura isolates.

### 2.3. Reverse Transcriptase-Polymerase Chain Reaction (RT-PCR)

Serotyping was conducted using multiplex RT-PCR, as described previously by Lanciotti et al. (1992) [11]. After confirming the serotype, RT-PCR to amplify the envelope (E) gene was performed again using serotype-specific sense and antisense primers designed on the premembrane coding region and nonstructural protein 1 coding region, respectively [8, 9]. RT-PCR products were purified using illustra ExoProStar (GE Healthcare, Little Chalfont, UK) and directly sequenced using the BigDye Terminator v1.1 (Applied Biosystems, Foster City, CA). Nucleotide sequences were elucidated using the ABI PRISM 310 Genetic Analyzer (Applied Biosystems, Foster City, CA).

### 2.4. Phylogenetic Analysis

A phylogenetic analysis of E gene sequences (1485 bp) was conducted as described previously [8]. More than 2000 dengue E gene sequences including all Surabaya isolates were retrieved from the GenBank database in each serotype for the construction of preliminary phylogenetic tree (data not shown). Then, several strains related to the Bangkalan strains were selected for further analyses using the Bayesian Markov chain Monte Carlo (MCMC) method, which is available in the Bayesian Evolutionary Analysis by Sampling Trees (BEAST) software package v1.5.3. This analysis used a relaxed uncorrelated lognormal molecular clock, a General Time Reversible + Γ model of nucleotide substitution for each codon position, and a Bayesian skyline coalescent model (five coalescent-interval groups). All chains were run for a sufficient length of time, with 100 million generations and multiple time points to ensure convergence, with 10% removed as burn-in. Genotype classification was performed by alignment with reference sequences [12].

## 3. Results

A total of 359 blood samples from suspected dengue patients were collected in Bangkalan, Madura Island, between 2012 and 2014. Seventeen samples (4.7%, 17/359) were positive for the isolation of DENV, as confirmed by immunostaining followed by RT-PCR; the results of both assays were consistent (Table 1). Of the 17 isolated viruses, 9 were classified as DENV-1 (52.9%), 7 as DENV-2 (41.2%), and 1 as DENV-3 (5.9%). DENV-4 was not isolated from our study subjects.

**Figure 1:**
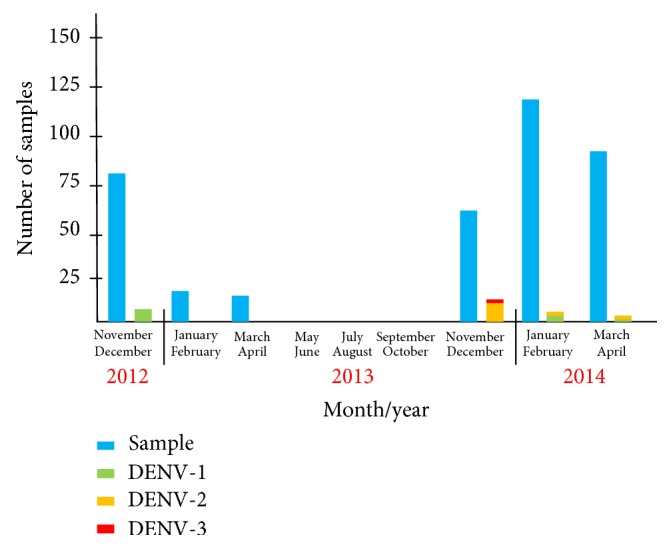
Monthly data on collected sera and isolated DENVs in Bangkalan between 2012 and 2014. The number of serum samples was shown in blue. Isolation numbers of DENV-1, DENV-2, and DENV-3 were shown in green, yellow, and red, respectively. DENV-4 was not isolated during the study period.

Figure 1 showed the monthly data of collected sera and isolated viruses. DENV-1 was the dominant (100%, 6/6) serotype in late 2012. The number of collected serum samples decreased between January and October 2013 due to the Indonesian dry season. DENV-2 was dominant between November and December 2013. DENV-1 then became dominant again between January and April 2014. DENV-3 was isolated in December 2013.

**Figure 2:**
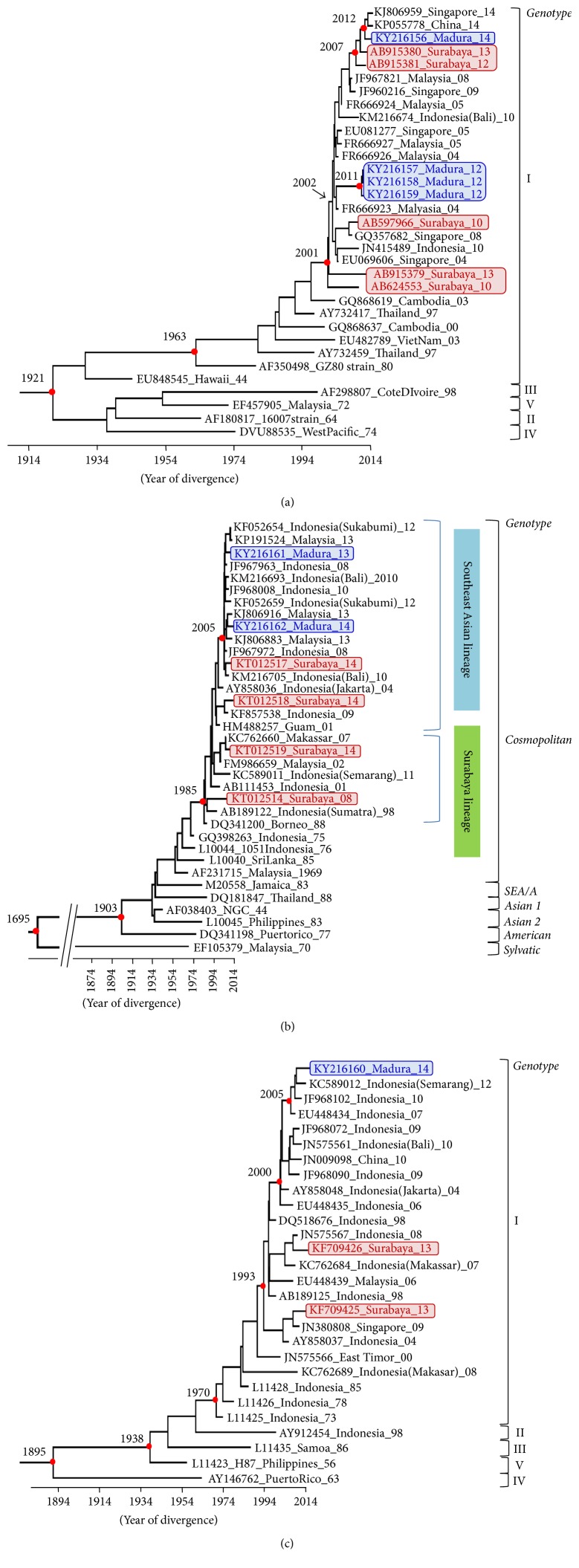
Phylogenetic trees of DENVs isolated in Bangkalan, Indonesia. Phylogenetic trees of DENV-1 (a), DENV-2 (b), and DENV-3 (c) were constructed using Bayesian Markov chain Monte Carlo (MCMC). The GenBank accession number, country/city, and year of isolation were shown in that order. The Bangkalan and Surabaya isolates were shown in blue and red, respectively. Filled red circles indicate statistical support at some key nodes. Posterior probability value > 0.9. Horizontal branches are drawn to the scale of the estimated year of divergence.

Seven isolates were randomly selected and subjected to a sequence analysis of the E coding region. The results of the phylogenetic study showed that all sequenced DENV-1 isolated in Madura Island were grouped into genotype I (Figure 2(a)). Three DENV-1 Madura Island isolates, KY216157_Madura_12, KY216157_Madura_12, and KY216159_Madura_12, formed a distinct clade from the other DENV-1 (posterior probability value = 1.0), while isolate KY216156_Madura_14 showed high nucleotide similarity to the Surabaya strains AB915381_Surabaya_12 and AB915380_Surabaya_13. Two DENV-2 isolates, KY216161_Madura_13 and KY216162_Madura_14, were grouped into the Cosmopolitan genotype (Figure 2(b)). They had a close phylogenetic relationship with KT012517_Surabaya_14. DENV-3 was genotype I (Figure 2(c)) and had a close phylogenetic relationship with JF968102_Indonesia_10 and KC589012_Indonesia(Semarang)_12.

## 4. Discussion

A total of 359 dengue-suspected samples were collected in the present study. The virus isolation rate was only 4.7% (17/359) from all patient sera, which was low. This low isolation rate was attributed to the possible misdiagnosis of dengue or the poor storage conditions of serum samples; there was no −80°C freezer in the hospital. In addition, patients in this area tended to visit hospital after four or five days of fever to save the medical cost. Most of the patients recruited in the study might not be in the peak of viremia when the blood was taken. Virus detection such as NS1 test or RT-PCR may improve the quality of samples and virus isolation rate.

The results of serotyping showed the presence of three DENV serotypes in Bangkalan, with DENV-1 as the predominant serotype, followed by DENV-2 and DENV-3. The predominant dengue serotype sequentially changed from DENV-1 to DENV-2 in November 2013 and to DENV-1 again in January 2014. This result indicated quick serotype shifts in this region. The Ministry of Health in East Java province reported that there were 397, 459, and 277 dengue cases in 2012, 2013, and 2014, respectively. The increase that occurred in the incidence of dengue in 2013 may have been due to the serotype shift from DENV-1 to DENV-2; however, it was not sufficient to conclude this hypothesis due to the possible underestimation of case number in the resource-limited area [13] and the few sequence results of the present study.

The viral transition pattern in Bangkalan was similar to that in Surabaya; a serotype shift from DENV-1 to DENV-2 occurred in July 2013 in Surabaya [9]. This shift suggested that viral circulation in Surabaya and Bangkalan was concomitant because workers from Bangkalan commute to and from Surabaya.

DENV-1 isolated in Madura Island was classified into genotype I, which is a typical genotype in Indonesia. DENV-1 Madura Island isolates in 2012 formed a distinct clade from the other DENV-1, indicating that this clade evolved uniquely in this area. KY216156_Madura_14 showed high nucleotide similarity to the Surabaya strains, suggesting viral introduction from Surabaya. All DENV-2 strains in Madura Island were grouped into the Cosmopolitan genotype. Bangkalan strains (KY216161_Madura_13 and KY216162_Madura_14) showed high nucleotide similarity to the Indonesian (KT012517_Surabaya_14) or Malaysian strains, which were previously classified to the Southeast Asian lineage [9]. This result supports the notion that the Southeast Asian lineage has the potential to become prevalent in various environments [9]. Both Bangkalan strains appeared to diverge in 2005, and the ancestral strain was indicated to be a Jakarta strain (AY858036_Indonesia (Jakarta)_04), suggesting viral introduction from Jakarta into Bangkalan in 2005. The DENV-3 strain (KY216160_Madura_14) clustered with Indonesian strains isolated in Bali, Jakarta, and Semarang, indicating a typical cluster in Indonesia. A Semarang strain showed the highest nucleotide similarity to the Madura Island isolate.

## 5. Conclusions

The present study is the first to provide molecular data on DENV in Bangkalan, Madura Island, Indonesia. The Madura isolates showed high nucleotide similarity to other Indonesian isolates, indicating frequent viral circulation in Indonesia. The results of the present study highlight the importance of continuous viral surveillance in dengue endemic areas in order to obtain a clearer understanding of the dynamics of DENV in Indonesia.

## Figures and Tables

**Table 1:** Information on dengue-infected patients from whom DENVs were isolated.

Number	Code of samples	Sex^*∗*^	Age^*∗∗*^	Day of onset	Serotype	GenBank accession number	Years
(1)	M43	M	16	5	DENV-1	—	2012
(2)	M48	F	10	4	DENV-1	KY216157	
(3)	M50	F	18	5	DENV-1	—	
(4)	M54	F	13	5	DENV-1	KY216158	
(5)	M56	M	7	5	DENV-1	—	
(6)	M60	M	16	5	DENV-1	KY216159	

(7)	M105	M	5	5	DENV-2	—	2013
(8)	M107	M	18	5	DENV-2	—	
(9)	M145	F	33	4	DENV-2	—	
(10)	M152	M	14	5	DENV-2	—	
(11)	M158	F	25	4	DENV-2	KY216161	
(12)	M163	M	11	5	DENV-3	KY216160	

(13)	M280	F	43	5	DENV-1	—	2014
(14)	M282	F	5	5	DENV-2	—	
(15)	M298	M	7	5	DENV-1	KY216156	
(16)	M340	M	34	5	DENV-2	KY216162	
(17)	M348	M	26	5	DENV-1	—	

^*∗*^F: female; M: male. ^*∗∗*^Age of patients in years.

## Abbreviations

MCMC: Bayesian Markov chain Monte Carlo
BEAST: Bayesian Evolutionary Analysis by Sampling Trees.
